# Serum uric acid concentration and blood pressure level in hemodialysis patients

**Published:** 2015-10-19

**Authors:** Viroj Wiwanitkit

**Affiliations:** ^1^Surin Rajabhat University, Surin, Thailand

**Keywords:** Blood pressure, Uric acid, Hemodialysis

**Implication for health policy/practice/research/medical education:**

Hemodialysis is usually required for the patients with end-stage renal failure. In the hemodialysis patients, abnormal blood pressure is commonly seen. In addition, the abnormalities of blood biochemical components are also common in hemodialysis patients. Recently much attention has been directed toward the blood pressure level and serum uric acid concentration in hemodialysis patients. However, the result of studies are contradictory and the linkage between observed serum uric acid and blood pressure requires further investigation.

Hemodialysis is usually required for the patients with end-stage renal failure. In the hemodialysis patients, abnormal blood pressure is commonly seen. In addition, the abnormalities of blood biochemical components are also common in hemodialysis patients. Recently much attention has been directed toward the blood pressure level and serum uric acid concentration in hemodialysis patients (1). In a study on 40 hemodialysis patients, Roozbeh et al showed the paradoxical correlation of high uric acid level with high systolic pressure, high mean arterial pressure (MAP) and wide pulse pressure. They found, these effects were independent of dialysis efficacy, dialysis duration and nutrition (1). In fact, the determined association is very interesting, however, there are many interesting issues to be addressed. Previously, Çağlı et al observed that, the increased serum uric acid is independently correlated with blood pressure variation in untreated essential hypertension individuals (2). This variation can be observed despite there is no hemodialysis (2). Therefore, in the present study, it is doubtful that the linkage between observed serum uric acid and blood pressure is accidentally related to dialysis. The variation of blood pressure might not be carefully assessed by Roozbeh et al. A visit to visit variability of blood pressure is known in the patients with advanced renal disease (3). Circadian blood pressure variation is not based on renal dysfunction alone (4). Having underlying diabetes mellitus increases the chance of highly variable blood pressure in dialysis patients (5). It is also observed that good nursing care can reduce the variability of blood pressure (6). Also, the use of different type and dosage of anti-hypertensive drug in each patient can also result in different blood pressure. In addition, the quality control of blood pressure measurement and uric acid determination should be discussed. With different tool and test, the reliability of the observed blood pressure and uric acid might be questionable. Different blood pressure monitoring and uric acid determination tools and techniques can result in different results. At least, the circadian rhythm in uremia (7) is well-known and has to be carefully considered in the present report. Additionally, there are several inferences, which must be controlled, on uric acid measurement in dialysis patients. For example, the extensively use drug, acetaminophen, is reported to have a great interference on uric acid determination in dialysis patients (8). In addition, there is a high pathophysiological variation of uric acid level in dialysis patients (9). The change of uric acid level in dialysis patients is very complex. The residual renal function is the main renal parameter relating to uric acid level (9). U-shaped, not straight, relationship between uric acid and residual renal function is reported (9). Therefore, studies regarding the linkage between observed serum uric acid and blood pressure requires further investigation.

## Author’s contribution

VW is the single author of the manuscript.

## Conflicts of interest

The author declared no competing interests.

## Ethical considerations

Ethical issues (including plagiarism, data fabrication, double publication) have been completely observed by the author.

## Funding/Support

None.
